# Investigating Non-Markovian Effects on Quantum Dynamics in Open Quantum Systems

**DOI:** 10.1021/acs.jctc.4c01632

**Published:** 2025-06-12

**Authors:** Mariia Ivanchenko, Peter L. Walters, Fei Wang

**Affiliations:** † Department of Chemistry and Biochemistry, 3298 George Mason University, 4400 University Drive, Fairfax, Virginia 22030, United States; ‡ Quantum Science and Engineering Center, 3298 George Mason University, 4400 University Drive, Fairfax, Virginia 22030, United States

## Abstract

The reduced description of the quantum dynamic processes in the condensed phase environment leads to the equation of motion with a memory kernel. Such a memory effect, termed non-Markovianity, presents more complex dynamics compared to its memoryless or Markovian counterpart, and many chemical systems have been demonstrated through numerical simulations to exhibit non-Markovian quantum dynamics. Explicitly how the memory impacts the dynamic process remains largely unexplored. In this work, we focus on ways to separate the non-Markovian contributions from the dynamics and study the non-Markovian effects. Specifically, we developed a rigorous procedure for mapping the exact non-Markovian quantum propagator to the Lindblad form. Consequently, it allows us to extract the negative decay rate from the Lindbladian that is the signature of the non-Markovianity. By including or excluding the negative rate in the time evolution, we can decisively pinpoint the influence of non-Markovianity on the system’s properties such as coherence, entanglement, and equilibrium state distribution. The understanding of such memory effects on the dynamic process suggests the possibility of leveraging non-Markovianity for quantum control.

## Graphical abstract

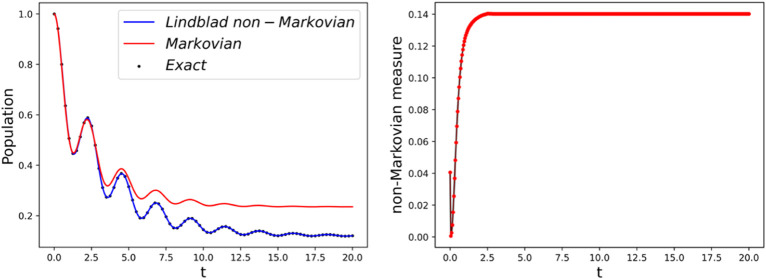

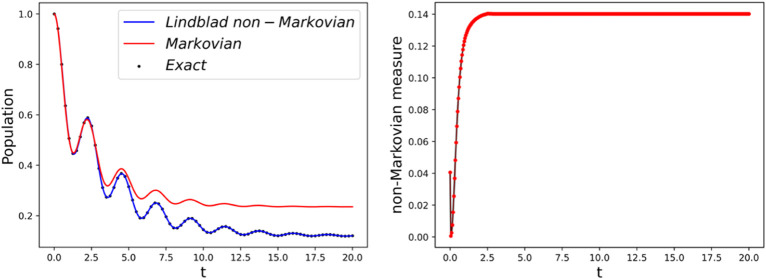

## 1 Introduction

The quantum dynamics of open quantum systems is the underpinnings of many charge and excitation energy transfer processes in the condensed phase environment.^1–8^ In the reduced density matrix (RDM) description, the dynamics of the quantum system often present as non-Markovian. Non-Markovianity refers to the fact that the past trajectory of the system has an influence on its current state and the future. This memory effect makes the simulation and analysis of non-Markovian quantum dynamics more demanding compared to Markovian ones. Intense efforts have been made to develop numerically exact methods for simulating non-Markovian quantum dynamics,^9–18^ and various non-Markovian witnesses and measures have been proposed to detect and quantify non-Markovianity in the quantum processes.^19^ On the other hand, however, a rigorous method that separates the non-Markovian contribution from the dynamics is still lacking, a method that would allow us to decisively investigate the memory effects on coherence, population dynamics, equilibrium states, etc. by turning on and off the non-Markovian contribution. In this work, we have developed a systematic approach to isolate the non-Markovian component from the dynamical equation and provided a useful framework for postcalculation analysis of the non-Markovian effects. We specifically focused on Feynman–Vernon’s influence functional formalism and the generalized Lindblad equation for the description of non-Markovian quantum dynamics. The organization of the Article is as follows. In Section 2, we discuss the definition of Markovian and non-Markovian quantum processes. In Section 3, we explore an equivalence relation used for defining the bona fide non-Markovian measure. In Section 4, we provide a step-by-step approach to mapping the exact non-Markovian quantum propagator to the Lindblad form. In Section 5, we discuss the characterizations of non-Markovian quantum processes using non-Markovian measures and the Bloch sphere representation. In Section 6, we provide simulation results for spin-boson dynamics and excitation energy transfer (EET) in model chromophore systems. In Section 7, we offer concluding remarks.

## 2 Markovian vs Non-Markovian Quantum Process

The time evolution of a RDM, ρ_*s*_, can be described by a propagator, ϕ_*t*_, formally known as a linear map or the so-called quantum operation or superoperator.

1 $$\rho_{s}\left(t\right)=\phi_{t}\left[\rho_{s}\left(0\right)\right]$$

Since such a linear map operates on a density matrix, which is positive semidefinitive (PSD), and returns another density matrix, it needs to be a positive map, i.e., transforming PSD matrices into PSD matrices. However, positivity (P) alone is not enough to justify a legitimate map. Suppose that ρ_*s*_ is the RDM of a larger quantum system ρ and that the only operation is ϕ_*t*_ acting on ρ_*s*_; it is essential that the end state ρ­(*t*) of the larger system is also a valid density matrix, i.e., PSD. This property of the map ϕ_*t*_ is called complete positivity (CP).^19,20^ At first glance, the check of the CP criterion requires the examination of the density matrices of any dimensions larger than the dimension of the system that ϕ_*t*_ is acting on. However, it can be shown that if ϕ_*t*_ is P for a larger system with the dimension of *d* ^2^, where *d* is the dimension of the system ϕ_*t*_ acts on, then the map is guaranteed to be CP. In addition, the Choi–Jamiolkowski isomorphism^21,22^ states the CP property of a map can be precisely checked by its corresponding Choi matrix, which will be elaborated in the next section.

Now consider CP maps ϕ_*t*_ and ϕ_*s*_ in which *t* ≥ *s* ≥ 0. We can define ϕ_*t,s*_ as ϕ_*t*_ ϕ_*s*_^–1^ so that ϕ_*t*_ *=ϕ*_*t,s*_ ϕ_*s*_, as shown in Figure 1. A quantum process is Markovian if and only if ϕ_*t,s*_ is CP for every *t* and *s*, which is termed CP-divisibility.^19,23^ Since this is both a necessary and sufficient condition, the violation of CP in ϕ_*t,s*_ will be used for the measure of non-Markovianity. The authors acknowledge that there are alternative definitions of non-Markovianity and its corresponding quantifications,^19,23^ such as distance measures and the one-shot two-state discrimination test. However, many of them are hard to compute in practice due to the optimization process involved. We specificity choose the CP divisibility in this work because of its computational tractability, especially for multistate quantum systems. Section 5.1 provides more details.

**Figure 1:**
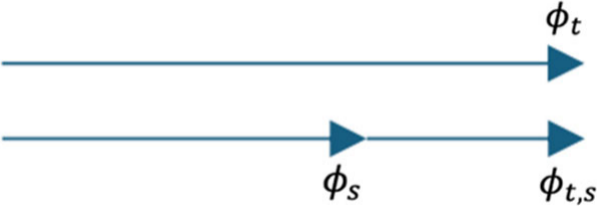
Linear maps of the quantum process.

The algebraic definitions of quantum Markovian and non-Markovian processes above have a connection to the physical setting. For system-bath dynamics, non-Markovianity is indicated by the building up of correlations between the system and bath. Therefore, the system’s (bath’s) trajectories are influenced by how the bath (system) has behaved, which incurs a memory effect on the dynamics. As pointed out by Pechukas,^24^ the linear map on the reduced dynamics of the correlated systems breaks CP, whereas for product states in which there are no system-bath correlations, CP can be preserved.

It is worth mentioning that a physically allowed linear map not only has to be a CP map but also is trace-preserving (TP), i.e., CPTP. However, if ϕ_*t*_ and ϕ_*s*_ are TP by construction, then ϕ_*t,s*_ is automatically TP as it is a composition of two TP maps, ϕ_*t*_ϕ_*s*_^–1^. Therefore, in many cases, it is only the CP criterion that needs to be checked when determining the non-Markovianity of a quantum process.

The CP-divisibility definition of Markovian processes conforms to our intuition; if the propagator of each of the small time steps is CP, then we can propagate the dynamics iteratively in time. Accordingly, a time-local master equation can be constructed. However, the reverse is not true. A time-local master equation is not guaranteed to be Markovian despite that, computationally, it can be solved by an iterative procedure. A typical example is the Redfield equation, which in some cases can produce negative populations.^25–27^ It should be noted that subtlety does exist in the literature as to what approximations to make to arrive at the Redfield equation. If the Born–Markov approximation is applied,^28,29^ then the Redfield equation can behave pathologically mentioned above as it breaks CP. If the additional secular approximation is applied,^30–32^ then such a Redfield equation is Markovian and can be mapped to the Gorini–Kossakowski–Sudarshan–Lindblad (GKSL) equation,^33,34^ which is the only form known to be a CPTP master equation.

## 3 Choi–Jamiolkowski Isomorphism

The Choi–Jamiolkowski isomorphism asserts a one-to-one correspondence between a linear map and a matrix, called the Choi matrix.^21,22^ Consider the maximally entangled state between the system that ϕ_*t,s*_ acts on and an auxiliary system.

2 $$|\Phi \rangle ≔\frac{1}{\sqrt{d}}\overset{d-1}{\underset{n=0}{\sum }}|n\rangle |n\rangle$$

The Choi matrix is defined as

3 $$\mathrm{Choi}\left(\phi_{t,s}\right)≔\left[\phi_{t,s}⊗I\right]|\Phi \rangle \langle \Phi |$$

Expanding it, we get

4 $$\mathrm{Choi}\left(\phi_{t,s}\right)=\underset{i,j}{\sum }\left\{\phi_{t,s}\left(|i\rangle \langle j|\right)\right\}⊗|i\rangle \langle j|$$

Each *i*,*j* block represents the effect of ϕ_*t*_,_*s*_ acting on the |*i*⟩⟨ *j*| “basis”. If ϕ_*t,s*_ is CPTP, then the Choi matrix is also a legitimate density matrix and vice versa. The non-Markovian quantum processes that violate the CP criterion will render the Choi matrix not a valid density matrix. Given that ϕ_*t,s*_ is trace preserving, some eigenvalues of such a Choi matrix will have negative values.^19,35^ This forms the basis for the detection and quantification of non-Markovianity in the quantum dynamic processes, which is further discussed in Section 5.

## 4 From the Exact Propagator to the Lindblad Form

### 4.1 Path Integral Formulation

Several numerically exact methods are available for constructing non-Markovian linear maps, such as QuAPI,^9,10^ QCPI,^11,12^ SMatPI,^36,37^ HEOM,^13^ ML-MCTDH,^14,15^ TNPI,^16,17^ etc. In this work, we use TNPI^16^ to generate such maps. We resort to a quantum system linearly coupled to its harmonic bath as our model system in the following discussion, as it has been shown to be widely applicable for simulating non-Markovian quantum dynamics in many condensed phase systems.^3–8^ In the Feynman’s path integral formulation, the time evolution of the RDM has the form

$$\rho_{s}\left(t\right)=\int Ds^{+}\int Ds^{-}\exp\left\{\frac{i}{\hbar }\left(S\left[s^{+}\right]-S\left[s^{-}\right]\right)\right\},\times IF\left[s^{+},s^{-}\right]\left\langle s_{0}^{+}\right|\rho_{s}\left(0\right)\left|s_{0}^{-}\right\rangle$$ 5

where

6 $$IF\left[s^{+},s^{-}\right]=\exp\left\{-\frac{1}{\hbar }\int_{0}^{t}dt^{\prime }\int_{0}^{t^{\prime }}dt^{\prime \prime }\left[s^{+}\left(t^{\prime }\right)-s^{-}\left(t^{\prime }\right)\right],\left[\alpha \left(t^{\prime }-t^{\prime \prime }\right)s^{+}\left(t^{\prime \prime }\right)-\alpha^{*}\left(t^{\prime }-t^{\prime \prime }\right)s^{-}\left(t^{\prime \prime }\right)\right]\right\}$$

is Feynman–Vernon’s influence functional,^2,38^ with *s* ^+^ and *s* ^–^ being the forward and backward paths, respectively. *S*[*s* ^+^] and *S*[*s* ^–^] are the action integrals of the free system, and ⟨*s*_0_^+^|ρ_*s*_(0)|*s*_0_^‑^⟩ is the initial state. The free propagator multiplied by the influence functional defines the map. The nonlocal memory kernel α­(*t*′ – *t*″) is the root of non-Markovianity and can be obtained from the bath response function^2^

7 $$\alpha \left(t\right)=\frac{1}{\pi }\int_{0}^{\infty }d\omega J\left(\omega \right)\left[\coth\left(\frac{\hbar \omega \beta }{2}\right)\cos\left(\omega t\right)-i\sin\left(\omega t\right)\right]$$

in which the spectral density *J*(ω) is defined as^39^

8 $$J\left(\omega \right)=\frac{\pi }{2}\underset{j}{\sum }\frac{c_{j}^{2}}{m_{j}\omega_{j}}\delta \left(\omega -\omega_{j}\right)$$

Here, ω_*j*_ is the bath frequency, and *c*_*j*_ is the coupling strength between the system and bath.

### 4.2 Converting the Propagator from the Path Integral Expression to the Lindblad form

To facilitate the analysis of non-Markovian effects in quantum dynamics, we provide in this section a rigorous procedure for deriving the Lindblad form from the non-Markovian propagator. Explicitly, the goal is that given ϕ_*t*_ in eq 1, the following form is constructed:

9 $$\overset{˙}{\rho_{s}}\left(t\right)=\Lambda_{t}\left[\rho_{s}\left(t\right)\right]≔-\frac{i}{\hbar }\left[H_{s}\left(t\right),\rho_{s}\left(t\right)\right]+\underset{k}{\sum }\gamma_{k}\left(t\right)\left(L_{k}\left(t\right)\rho_{s}\left(t\right)L_{k}^{†}\left(t\right)-\frac{1}{2}\left\{L_{k}^{†}\left(t\right)L_{k}\left(t\right),\rho_{s}\left(t\right)\right\}\right)$$

where {} is the anticommutator. Unlike the conventional Lindblad equation, i.e., the GKSL equation, the decay rates γ_*k*_ (*t*) in eq 9 can have negative values, which indicates non-Markovianity.

We decomposed the task into three steps. First, for a Hilbert space of dimension *d*, we express ϕ_*t*_ in the orthonormal basis of Hermitian operators {*G*_*m*_} such that

10 $$G_{0}=\frac{I}{\sqrt{d}};G_{m}=G_{m}^{†};\mathrm{Tr}\left[G_{m}G_{n}\right]=\delta_{mn}$$

in which I is the identity operator. There are a total of *N* = *d* ^2^ such basis operators. For a two-level system, the operators that satisfy eq 10 are Pauli matrices (and identity), and for a three-level system these are the Gell–Mann matrices, which are the analogues of the Pauli matrices. The *n*-level generalization can also be constructed,^40,41^ the matrices of which are Hermitian, traceless, and orthogonal. They are the so-called generalized Gell–Mann matrices and constitute the generators of the su(n) group.

To relate a linear map ϕ_*t*_ to the corresponding Λ_*t*_, we define

11 $$F_{kl}\left(t\right)≔\mathrm{Tr}\left[G_{k}\phi_{t}\left(G_{l}\right)\right]$$

which, by analogy with the bra-ket notation, puts the superoperator ϕ_*t*_ in the {*G*_*m*_} basis representation.

Because ρ_*s*_(*t*) = ϕ_*t*_[ρ_*s*_(0)], then *ρ̇*_*s*_(*t*) = *ϕ̇*_*t*_[ρ_*s*_(0)] = *ϕ̇*_*t*_{ϕ_*t*_^‑1^[ρ_*t*_(*t*)]}. By comparing it with *ρ̇*_*s*_(*t*) = Λ_*t*_ [ρ_*s*_(*t*)], we get Λ_*t*_ = *ϕ̇*_*t*_ϕ_*t*_^–1^. If the inverse of the superpropagator does not exit, then pseudoinverse can be employed.

Therefore, we can calculate

12 $$B\left(t\right)=Ḟ\left(t\right)F^{-1}\left(t\right)$$

in which

13 $$B_{kl}\left(t\right)≔\mathrm{Tr}\left[G_{k}\Lambda_{t}\left(G_{l}\right)\right]$$

being the superoperator Λ_*t*_ in the {*G*_*m*_} basis.

Second, we find the operator-sum representation of the Λ_*t*_ by computing^42^

14 $$D_{ij}\left(t\right)≔\overset{N-1}{\underset{k,l=0}{\sum }}B_{kl}\left(t\right)\mathrm{Tr}\left[G_{l}G_{i}G_{k}G_{j}\right]$$

Then,

15 $$\Lambda_{t}\left[\rho_{s}\left(t\right)\right]=\overset{N-1}{\underset{k,l=0}{\sum }}D_{ij}\left(t\right)G_{i}\rho_{s}\left(t\right)G_{j}$$

which closely resembles the second term in the Lindblad form. *D*_*ij*_(*t*) is the Choi matrix representation of the superoperator Λ_*t*_ and it is easy to show that *D*_*ij*_(*t*) is Hermitian.

Third, we express Λ_t_(ρ) in a canonical Lindblad form.^1,43^ We may define

16 $$A\left(t\right)≔\frac{1}{2}\frac{D_{00}\left(t\right)}{d}+\overset{N-1}{\underset{i=1}{\sum }}\frac{D_{i0}\left(t\right)}{\sqrt{d}}G_{i}$$

Then the system Hamiltonian can be identified as

17 $$H_{s}\left(t\right)≔\frac{i}{2}\hbar \left(A\left(t\right)-A^{†}\left(t\right)\right)$$

Subsequently, the dynamical equation can be expressed as

18 $$\overset{˙}{\rho_{s}}\left(t\right)=-\frac{i}{\hbar }\left[H_{s}\left(t\right),\rho_{s}\left(t\right)\right]+\overset{N-1}{\underset{i,j=1}{\sum }}D_{ij}\left(t\right)\left(G_{i}\rho_{s}\left(t\right)G_{j}-\frac{1}{2}\left\{G_{j}G_{i},\rho_{s}\left(t\right)\right\}\right)$$

where *D*_*ij*_(*t*) (*i*, *j* ≠ 0) is the decoherence matrix.

As *D*_*ij*_(*t*) (*i*, *j* ≠ 0) is Hermitian, it can be diagonalized

19 $$D_{ij}\left(t\right)=\overset{N-1}{\underset{k=1}{\sum }}U_{ik}\left(t\right)\gamma_{k}\left(t\right)U_{jk}^{*}\left(t\right)$$

where γ_*k*_(*t*) are the eigenvalues of *D*_*ij*_(*t*) (*i*, *j* ≠ 0).

The Lindblad operators are defined as

20 $$L_{k}\left(t\right)≔\overset{N-1}{\underset{i=1}{\sum }}U_{ik}\left(t\right)G_{i}$$

which recovers the form of eq 9. We call the Lindblad form thus constructed the *generalized* Lindblad equation.

It should be noted that for simulating a specific dynamical process, the Lindblad equation is not unique. It is invariant under unitary and inhomogeneous transformations.^44^ However, this does not pose an issue for the above approach. With the su(n) generators as the basis set, the Lindblad equation is uniquely constructed, and the eigenvalues γ_*k*_ (*t*) from the diagonalization of *D*_*ij*_(*t*) are uniquely defined. The γ_*k*_(*t*) produced in this way directly correlates with the non-Markovian measure discussed in Section 5.1.

## 5 Characterization of Non-Markovianity

### 5.1 Non-Markovian Measures

Although many theoretical methods have been proposed to detect non-Markovianity, most of them are classified as non-Markovian witnesses.^35,45–51^ A witness may detect non-Markovianity only in certain cases, whereas a non-Markovian measure^35,43,52–54^ is a bona fide quantifier that is nonzero if and only if non-Markovian dynamics is present. Some measures such as those based on optimization^52,53^ are difficult to compute in practice, whereas the ones based on the violation of the CP^35^ and on the negative decay rate^43^ are amenable to calculations within the framework of the Lindblad equation.

#### 5.1.1 RHP Measure

We first introduce the RHP (Rivas, Huegla, and Plenio) measure,^35^ which is based the quantification of how much the dynamics deviates from CP. As indicated in Section 3, the Choi matrix^21,22^ of the Markovian short-time propagator is CP and the sum of the absolute value of its eigenvalues is exactly one; on the contrary, such a sum for the Choi matrix of the non-Markovian short-time propagator is larger than one.^19,35^ Since the short-time propagator can be expressed from the Lindblad superoperator as $I+\varepsilon \Lambda_{t}$ , with ε being an arbitrarily small value, then Choi matrix associated with this short-time propagator is

21 $$\left[I+\varepsilon \left(\Lambda_{t}⊗I\right)\right]|\Phi \rangle \langle \Phi |$$

The sum of the absolute value of the eigenvalues can be calculated by the trace-norm. Therefore,

22 $${∥\left[I+\varepsilon \left(\Lambda_{t}⊗I\right)\right]|\Phi \rangle \langle \Phi |∥}_{1}\{\begin{array}{ll} =1 & \mathrm{if Markovian}\\ >1 & \mathrm{if non‐Markovian} \end{array}$$

where ∥•∥_1_ is the trace norm or the Schatten 1-norm defined as

23 $${∥A∥}_{1}≔\mathrm{Tr}\sqrt{AA^{†}}$$

which is equal to the sum of the singular values of *A*. The RHP measure^35^ quantifies the non-Markovianity by defining a function *g*(*t*) such that

24 $$g\left(t\right)≔\underset{\varepsilon \to 0^{+}}{\lim}\frac{{∥\left[I+\varepsilon \left(\Lambda_{t}⊗I\right)\right]|\Phi \rangle \langle \Phi |∥}_{1}-1}{\varepsilon }$$

#### 5.1.2 Decay Rate Measure

It has been shown that the negative value of the decay rate γ_*k*_(*t*) in the Lindblad dissipator can also serve as a true measure of non-Markovianity.^43^ Whereas a positive decay rate indicates decoherence, a negative rate can be perceived as the system recohering. Specifically, the non-Markovianity of a specific Lindblad channel can be quantified by

25 $$f_{k}\left(t\right)≔\max\left[0,-\gamma_{k}\left(t\right)\right]\geq 0$$

whereas the non-Markovianity for the dynamics at time *t* can be calculated by

26 $$f\left(t\right)≔\overset{N-1}{\underset{k=1}{\sum }}f_{k}\left(t\right)$$

The decay rate measure provides a way to find the *conjugate* Markovian dynamics from the non-Markovian dynamics by eliminating the negative values of the decay rates in the generalized Lindblad equation. The equivalence between the decay rate measure and the RHP measure has been elegantly proved, stating that^43^

27 $$f\left(t\right)=\frac{d}{2}g\left(t\right)$$

where *d* is the dimension of the state space. The advantage of using the Lindblad form is that one can define a “non-Markov index” as the number of negative decay rates,^43^ quantifying the dimension of space permeated by non-Markovian dynamics. In addition, it has been demonstrated that the minimal rate of isotropic noise that must be added to produce Markovian dynamics is given by the most negative decay rate.^43,52^

### 5.2 Bloch Sphere Representation

The Bloch sphere can conveniently describe the qubit state and its dynamical process. The density matrix of a qubit can be expressed as

28 $$\rho =\frac{\left(I+\mathrm{r⃗}\cdot \mathrm{\sigma ⃗}\right)}{2}$$

where *r⃗* is a real three-component vector and σ⃗ are the Pauli matrices. Any trace-preserving quantum operation on a qubit corresponds to an affine transformation,

29 $${\mathrm{r⃗}}^{\prime }=M\mathrm{r⃗}+\mathrm{C⃗}$$

where *C⃗* is a constant vector that accounts for translation of the Bloch sphere and *M* is a 3 × 3 real matrix. The polar decomposition of the matrix *M* is given by

30 M=OS

where *O* is a real orthogonal matrix with determinant 1 and *S* is a real symmetric matrix.^20^ Therefore, the linear transformation *M* represents first a stretching and/or compression (determined by *S*) of the Bloch sphere along the principal axes, and then a rotation (determined by *O*). We can clearly visualize the difference between Markovian and non-Markovian processes for qubit dynamics and assess the non-Markovian contribution to the linear transformation and to the translation.

The Bloch volume evolves according to *V*(*t*) = *V*(0)­det *M*(*t*).^49^ Hence, the increase in the Bloch volume is a witness to the non-Markovianity. It can be shown that

31 $$\frac{d}{dt}\det M\left(t\right)=\mathrm{Tr}\left[B\left(t\right)\right]\det M\left(t\right)$$

where *B*(*t*) is given in eq 13. The Bloch volume increase implies Tr­[*B*(*t*)] > 0. Moreover,^43^

32 $$\mathrm{Tr}\left[B\left(t\right)\right]=-d\overset{N-1}{\underset{k=1}{\sum }}\gamma_{k}\left(t\right)$$

pointing to the benefit of working with the Lindblad form. The translational vector *C⃗* can be accessed by

33 $$C_{i}=\mathrm{Tr}\left[G_{i}\phi_{t}\left(I\right)\right]/d,i>0$$

in which ϕ_*t*_ could be the propagator for the non-Markovian dynamics or its conjugate Markovian one.

## 6 Results and Discussions

In what follows, we present the simulation results for two model systems. One is the spin-boson dynamics, and the other is excitation energy transfer (EET) in the model chromophore dimer.

### 6.1 Spin-Boson Dynamics

In the spin-boson model, the system-bath Hamiltonian is given by

34 $$H=\hbar \Omega \sigma_{x}+\hbar \varepsilon \sigma_{z}+\underset{j}{\sum }\left[\frac{{p_{j}}^{2}}{2m_{j}}+\frac{1}{2}m_{j}{\omega_{j}}^{2}{\left(x_{j}-\frac{c_{j}\sigma_{z}}{m_{j}{\omega_{j}}^{2}}\right)}^{2}\right]$$

where *p*_*j*_ and *x*_*j*_ denote the bath’s momentum and position, respectively, and *c*_*j*_ denotes the system-bath coupling strength. We choose the spectral density to have the Ohmic form

35 $$J\left(\omega \right)=\frac{\pi }{2}\hbar \xi \omega e^{-\omega /\omega_{c}}$$

which gives a continuous version of eq 8. The dimensionless ξ is the Kondo parameter that determines the strength of the system-bath coupling, and ω_*c*_ is the cutoff frequency of the bath. Our simulations and analysis explore three parameter regimes. Figures 2–4 have the parameters of Ω = 1, β = 5, ξ = 0.1, ω_*c*_ = 7.5, and *ε* = 0, which show damped oscillation of a symmetric two-state system. Figures 5 have the parameters of Ω = 1, β=5, ξ = 0.1, ω_*c*_ = 7.5, and *ε* = 1, which show damped oscillation of an asymmetric two-state system. Figures 8–10 have the parameters of Ω = 1, β = 5, ξ = 0.5, ω_*c*_ = 7.5, and *ε* = 1, which show the dissipative decay of an asymmetric two-state system. In each parameter set, it displaces six items: (1) the matching results of the generalized Lindblad equation with the exact benchmark, (2) the separation of the conjugate Markovian dynamics from its corresponding non-Markovian one, (3) the decay rates, (4) the matching of the decay rate measure and the RHP measure, (5) the difference in the translational vector between non-Markovian and its conjugate Markovian dynamics, and (6) the comparison of the Bloch sphere dynamics between the non-Markovian and its conjugate Markovian process.

**Figure 2:**
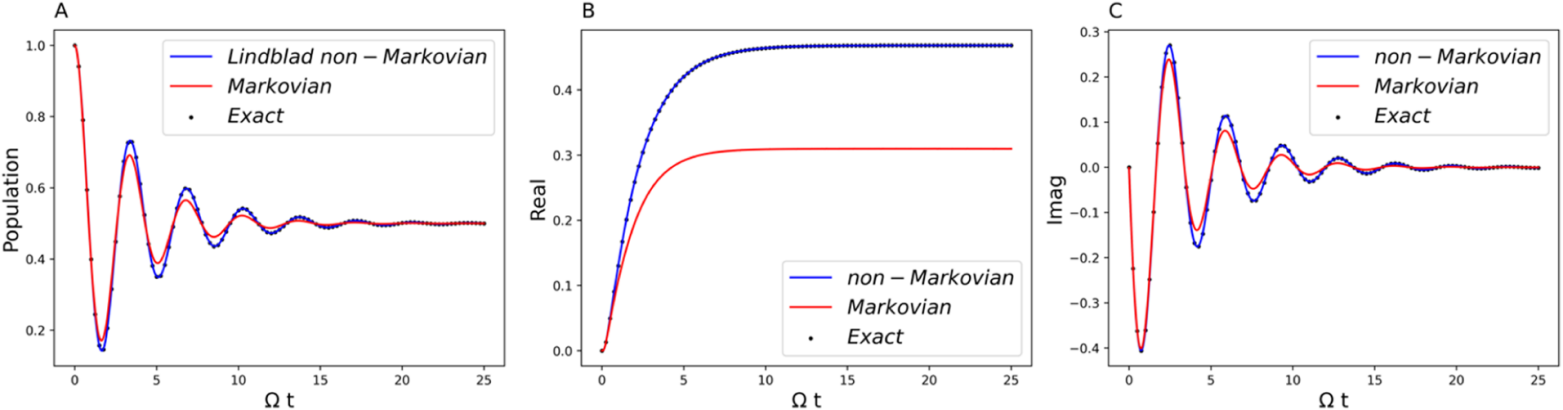
Comparison of the generalized Lindblad equation result (blue curve) with the exact benchmark (black dot) and the comparison of the non-Markovian (blue curve) and Markovian (red curve) dynamics. Parameters are Ω = 1 and *ε* = 0 for the system and β = 5, ξ = 0.1, and ω_*c*_ = 7.5 for the Ohmic bath. (A) Population dynamics of the symmetric two-state system. (B) The real part of the off-diagonal element of the RDM. (C) The imaginary part of the off-diagonal element of RDM.

**Figure 3:**
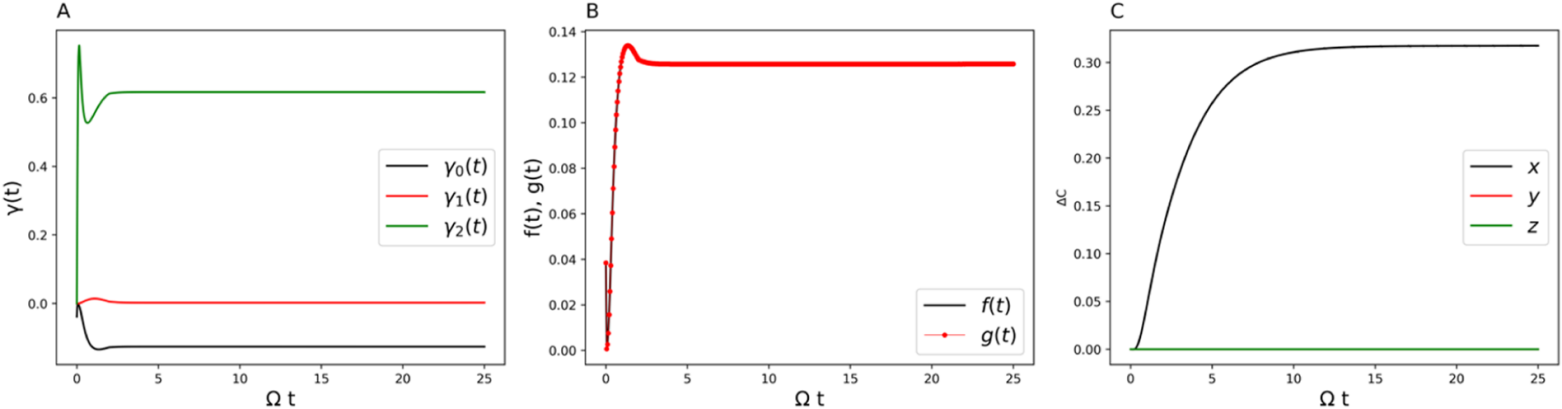
(A) Time-dependent γ coefficients (decay rates). (B) Comparison of the decay rate measure *f*(*t*) with the RHP measure *g*(*t*) for the two-state system. (C) Non-Markovian and Markovian comparison of the displacement of the center of the Bloch sphere over time. Parameters are Ω = 1 and *ε* = 0 for the system and β = 5, ξ = 0.1, and ω_*c*_ = 7.5 for the Ohmic bath.

**Figure 4:**
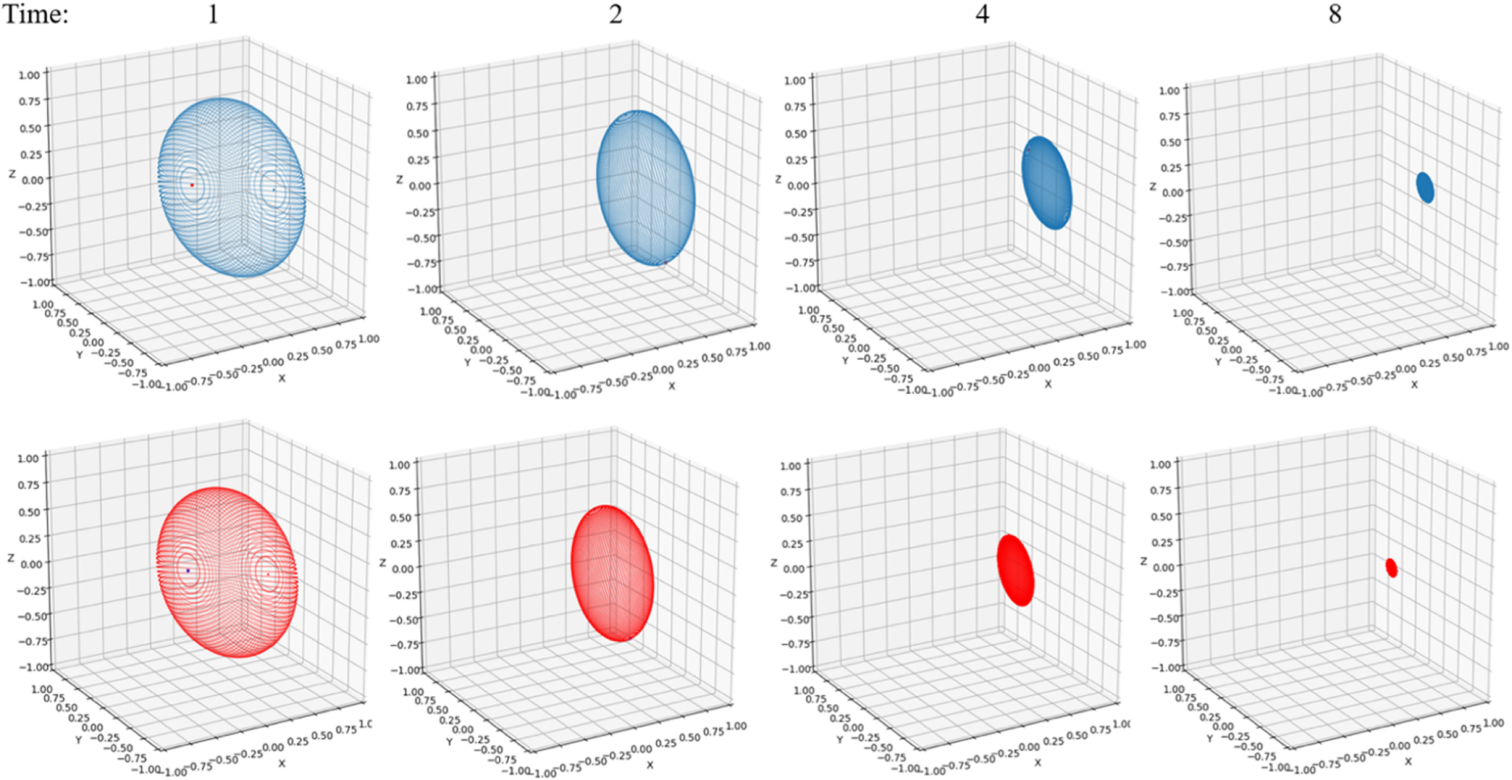
Bloch sphere under non-Markovian (blue) and Markovian (red) evolution at *t* = 1, *t* = 2, *t* = 4, and *t* = 8. Parameters are Ω = 1 and *ε* = 0 for the system and β = 5, ξ = 0.1, and ω_*c*_ = 7.5 for the Ohmic bath.

**Figure 5:**
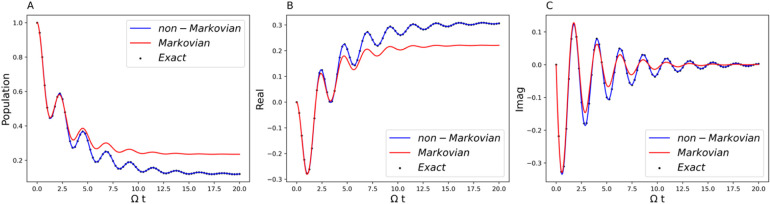
Comparison of the generalized Lindblad equation result (blue curve) with the exact benchmark (black dot) and the comparison of the non-Markovian (blue curve) and Markovian (red curve) dynamics. Parameters are Ω = 1 and *ε* = 1 for the system and β = 5, ξ = 0.1, and ω_*c*_ = 7.5 for the Ohmic bath. (A) Population dynamics of the symmetric two-state system. (B) The real part of the off-diagonal element of the RDM. (C) The imaginary part of the off-diagonal element of RDM.

**Figure 6:**
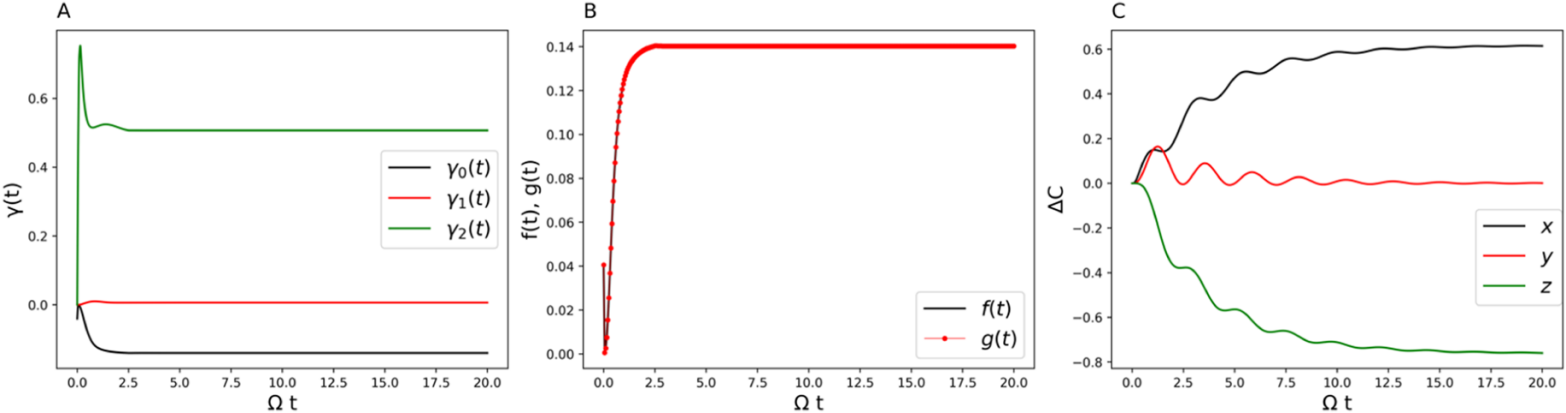
(A) Time-dependent γ coefficients (decay rates). (B) Comparison of the decay rate measure *f*(*t*) with the RHP measure *g*(*t*) for the two-state system. (C) Non-Markovian and Markovian comparison of the displacement of the center of the Bloch sphere over time. Parameters are Ω = 1 and *ε* = 1 for the system and β = 5, ξ = 0.1, and ω_*c*_ = 7.5 for the Ohmic bath.

**Figure 7:**
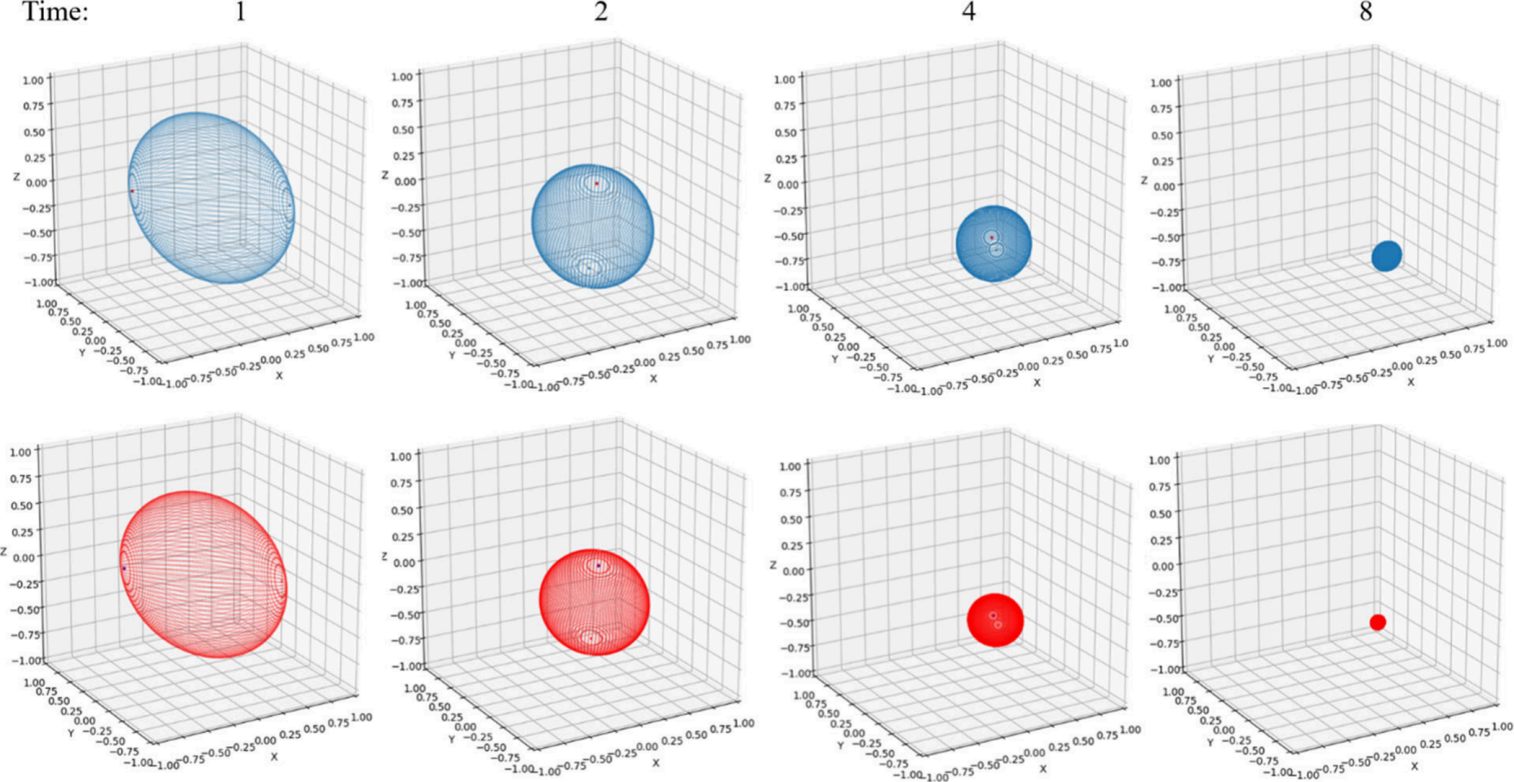
Bloch sphere under non-Markovian (blue) and Markovian (red) evolution at *t* = 1, *t* = 2, *t* = 4, and *t* = 8. Parameters are Ω = 1 and *ε* = 1 for the system and β = 5, ξ = 0.1, and ω_*c*_ = 7.5 for the Ohmic bath.

**Figure 8:**
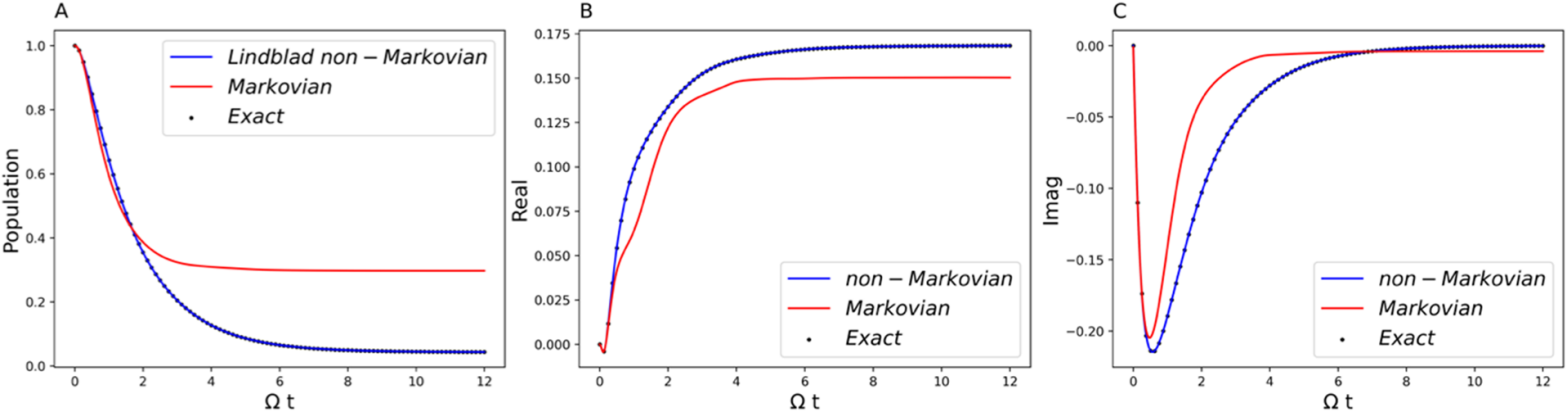
Comparison of the generalized Lindblad equation result (blue curve) with the exact benchmark (black dot) and comparison of the non-Markovian (blue curve) and Markovian (red curve) dynamics. Parameters are Ω = 1 and *ε* = 1 for the system and β = 5, ξ = 0.5, and ω_*c*_ = 7.5 for the Ohmic bath. (A) Population dynamics of the symmetric two-state system. (B) The real part of the off-diagonal element of the RDM. (C) The imaginary part of the off-diagonal element of RDM.

**Figure 9:**
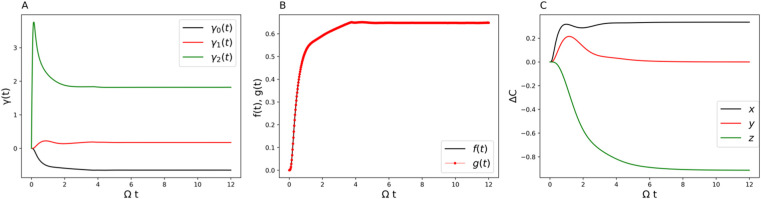
(A) Time-dependent γ coefficients (decay rates). (B) Comparison of the decay rate measure *f*(*t*) with the RHP measure *g*(*t*) for the two-state system. (C) Non-Markovian and Markovian comparison of the displacement of the center of the Bloch sphere over time. Parameters are Ω = 1 and *ε* = 1 for the system and β = 5, ξ = 0.5, and ω_*c*_ = 7.5 for the Ohmic bath.

**Figure 10:**
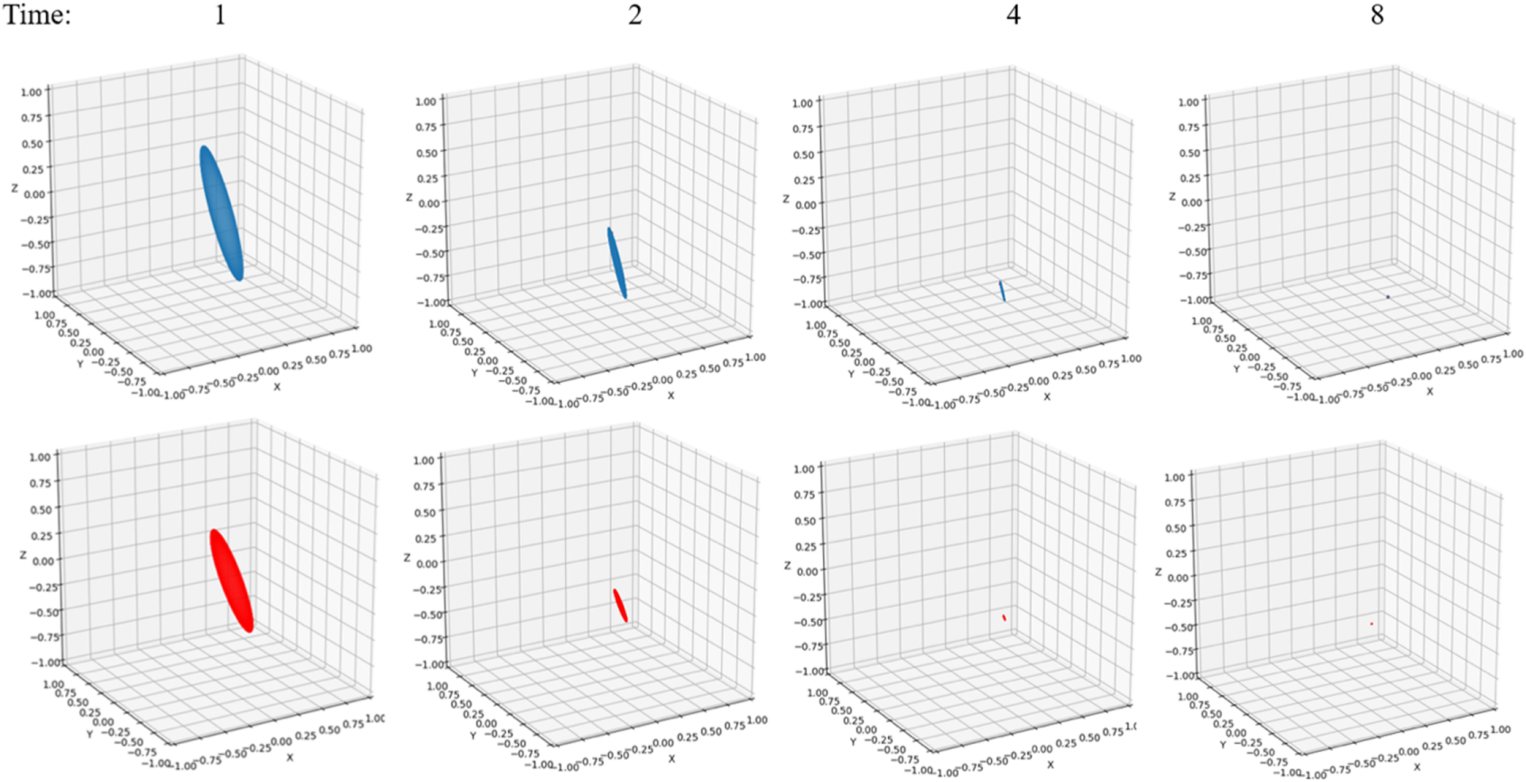
Bloch sphere under non-Markovian (blue) and Markovian (red) evolution at *t* = 1, *t* = 2, *t* = 4, and *t* = 8. Parameters are Ω = 1 and *ε* = 1 for the system and β = 5, ξ = 0.5, and ω_*c*_ = 7.5 for the Ohmic bath.

Figures 2, 5, and 8 clearly demonstrate that the Lindblad form (blue curve) constructed by the procedures outlined in Section 4.2 can produce exactly the correct dynamical behavior (black dot), confirming the reliability of using the canonical Lindblad formalism to simulate non-Markovian quantum dynamics. The exact benchmark simulations are obtained from the TNPI^16^ method. Figures 3A, 6A, and 9A show the decay rates γ_*k*_ (*t*) in the Lindblad equation. They are time-dependent, and the negative values indicate non-Markovianity. The perfect match of the decay rate measure with the RHP measure is shown in Figures 3B, 6B, and 9B. It clearly points to the robustness of using the Lindblad form to analyze non-Markovian quantum dynamics. In what follows, we will give a thorough analysis of the results for parameter set 1 and briefly point to the similarities and differences for parameter sets 2 and 3.

Figure 2 shows the separation of Markovian dynamics (red curve) from the fully non-Markovian dynamics (blue curve) by setting the negative values of the decay rate to zero. The plots in Figure 2A–C are the population dynamics, the real part of the off-diagonal element of the RDM, and the imaginary part of the off-diagonal of the RDM, respectively. Two distinctive features emerge from the comparison. First, the non-Markovian bath makes the coherence less degraded compared to the Markovian bath, as can be seen in the larger amplitude of the oscillation in the non-Markovian case. In other words, non-Markovianity has the benefit of hedging against decoherence and dissipation. This can also be seen from Figure 2B and C, the coherence part of the RDM. The imaginary part of the off-diagonal element of the RDM is related to the time derivative of the population,^55^ and therefore Figure 2C correctly correlates with the behavior in panel A. The second feature arises from the observation of Figure 2B. The real part of the off-diagonal element of the RDM is equal to one-half of the population difference between the two eigenstates.^56^ The non-Markovian bath has the effect of opening up the energy gap and therefore leading to the possibility of tuning the system’s “effective” energy levels by leveraging non-Markovianity. Since the real part of the off-diagonal stabilizes at a larger value in the non-Markovian case, it has the effect of coherence trapping.^57^

Figure 3A shows the decay rates from the Lindblad dissipators. For parameter set 1, only one Lindblad channel contributes to the non-Markovian behavior. Figure 3B shows the exact matching of the RHP measure and the decay rate measure, demonstrating once again the numerical reliability of using the generalized Lindblad equation to describe non-Markovian quantum dynamics. Figure 3C gives the difference in the translational vector *C⃗* in eq 29 between the non-Markovian dynamics and its conjugate Markovian dynamics. The difference lies only in the *x*-component, which shows that the non-Markovianity is solely encoded in one-dimensional translational motion among the three.

Figure 4 gives a pictorial representation of the non-Markovian and Markovian time evolution by plotting the Bloch sphere change over time. As discussed in Section 5.2, the rate of change is determined by the *M* matrix in eq 31. It can be clearly observed that the Markovian dynamics suffers more from the dissipation and decoherence from the bath, with the Bloch volume shrinking at a faster rate. Both spheres will eventually collapse to a point that has zero value in the *y*- and *z*-components and a finite value in the *x*-component. The population reaches the 50–50 equilibrium state, indicating the *z* component goes to zero. The imaginary part of the off-diagonal element approaches zero, indicating the y component is zero. Only the real part of the off-diagonal element reaches a finite value, which contributes to the σ_*x*_ component in eq 26. These exactly match the behaviors in Figure 2B and 3C. It is interesting to compare this non-Markovian feature with the witness defined by the Bloch volume increase (eqs 31 and 32). We can see from Figure 3A that the sum of these decay rates gives a positive value, hence the Bloch volume keeps decreasing over time. This is also true for Figure 6A and 9A by coincidence. It raises caution about the use of the Bloch volume increase^49^ (a sign of information back-flow) as evidence of non-Markovianity.

Figure 5 demonstrates the difference between non-Markovian dynamics and its conjugate Markovian dynamics for an asymmetric two-state system. Besides the coherence protection and coherence trapping features observed for the symmetric system, Figure 5A clearly indicates that the non-Markovian bath alters the equilibrium constant. Particularly in this case, it drives the reaction to a higher percentage yield. Figure 8A shows the same trend with a different parameter set. Therefore, another usefulness of the non-Markovian bath arises from its ability to shift the chemical equilibrium. Figure 6C and 7 restate the properties of equilibrium shift (*z*-component) and the coherence trapping (*x*-component) by the non-Markovian bath.

Whereas Figures 2–7 show damped oscillations, Figures 8–10 show pure dissipative decay. They reveal the same features thus discussed. It is interesting to note that, in this example, the Bloch sphere begins to flatten and becomes needle-like with the time evolution (Figure 10), indicating that the propagator loses dimensions, and consequently the inverse matrix, *F* ^–1^ (*t*) in eq 12, is not well-defined in those times. However, with pseudoinverse, the correct dynamics can be perfectly reproduced with the generalized Lindblad equation (Figure 8).

### 6.2 EET in the Chromophore Dimer

In this example, we consider the exciton dynamics in the model chromophore dimer system. Restricting the system to the single-exciton Frenkel subspace |01⟩ and |10⟩, we have essentially a two-level system. The system-bath Hamiltonian is given by

36 $$H=\hbar \Omega \sigma_{x}+\hbar \varepsilon \sigma_{z}+\underset{j,b}{\sum }\frac{{p_{jb}}^{2}}{2m_{jb}}+\frac{1}{2}m_{jb}{\omega_{jb}}^{2}{\left(x_{jb}-\frac{c_{jb}{\mathrm{V̂}}_{j}}{m_{jb}{\omega_{jb}}^{2}}\right)}^{2}$$

in which the *b* ^th^ vibrational mode associated with the *j* ^th^ monomer has the frequency of ω_*jb*_ and the coupling strength of *c*_*jb*_. *V̂*_*j*_ is the projection operator on the excited state of monomer *j*. Following the work of Ishizaki and Fleming,^58^ we choose the system parameters to be ℏΩ = 100 cm^–1^ and *ℏε* = 50 cm^–1^ and the spectral density to have the Drude form,

37 $$J\left(\omega \right)=2\lambda \gamma \frac{\omega }{\omega^{2}+\gamma^{2}}$$

with the reorganization energy λ = 20 cm^–1^ and the cutoff frequency γ = 53.08 cm^–1^. The temperature is at *T* = 300 K. The system is initially in one localized excitonic state.

The results are shown in Figures 11–13. Besides the common features observed in the spin-boson model, it is of interest to note that coherence oscillation observed in Figure 11 is indeed the entanglement, since only the single exciton states | 01⟩ and |10⟩ are considered. These calculations show that the non-Markovian bath has the effect of maintaining entanglement longer.

**Figure 11:**
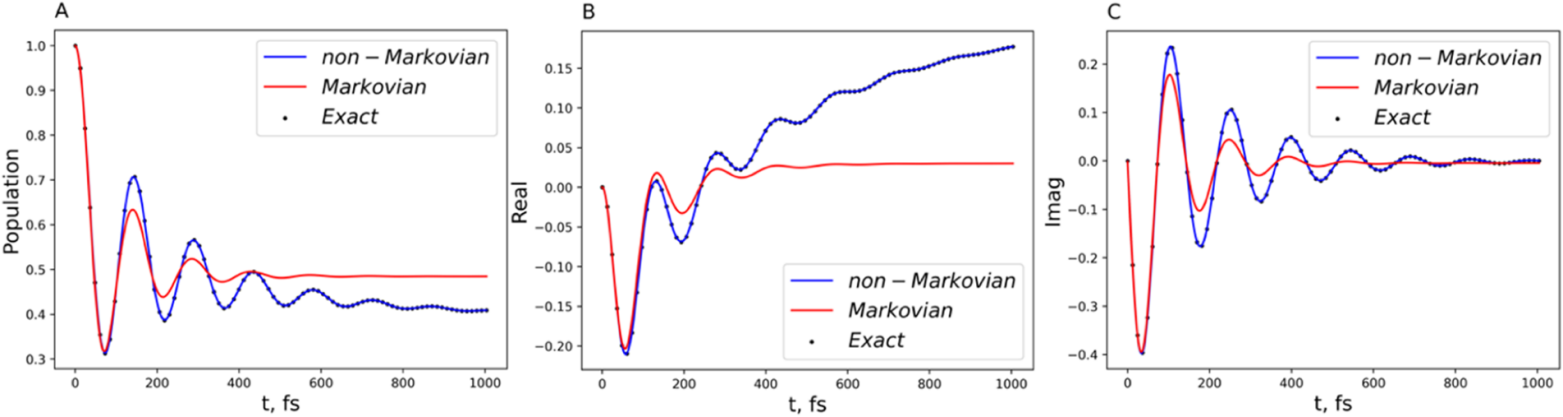
Comparison of the generalized Lindblad equation result (blue curve) with the exact benchmark (black dot) and comparison of the non-Markovian (blue curve) and Markovian (red curve) dynamics. Parameters are ℏΩ = 100 cm^–1^ and ℏ*ε* = 50 cm^–1^ for the system and λ = 20 cm^–1^, γ = 53.08 cm^–1^, and *T* = 300 K for the Drude bath. (A) Population dynamics of the symmetric two-state system. (B) The real part of the off-diagonal element of the RDM. (C) The imaginary part of the off-diagonal element of RDM.

**12:**
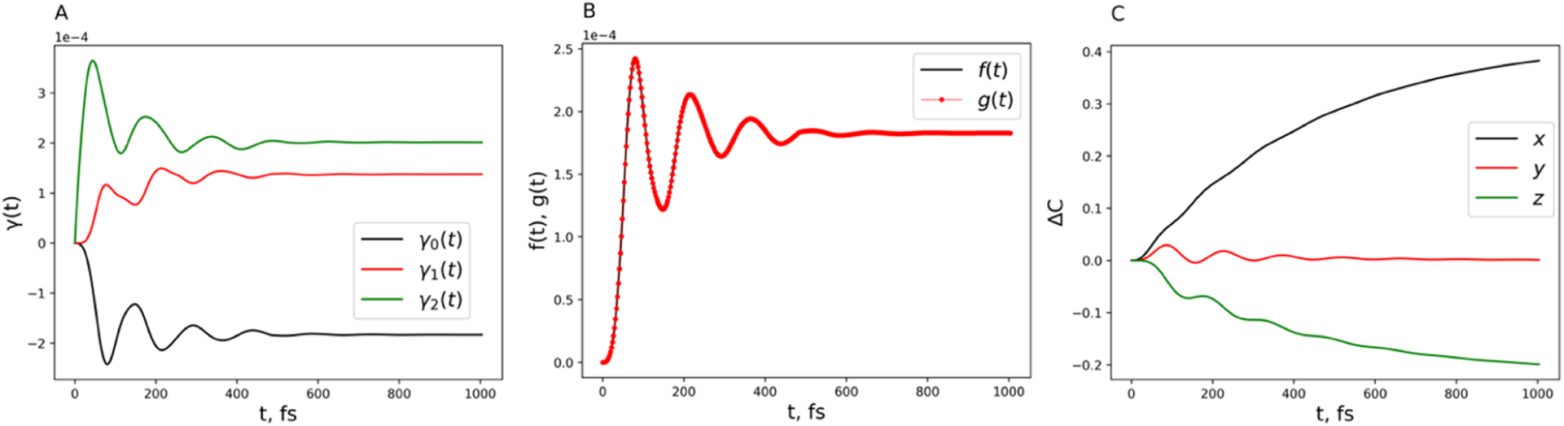
(A) Time-dependent γ coefficients (decay rates). (B) Comparison of the decay rate measure *f*(*t*) with the RHP measure *g*(*t*) for the two-state system. (C) Non-Markovian and Markovian comparison of the displacement of the center of the Bloch sphere over time. Parameters are ℏΩ = 100 cm^–1^ and ℏ*ε* = 50 cm^–1^ for the system and λ = 20 cm^–1^, γ = 53.08 cm^–1^, and *T* = 300 K for the Drude bath.

**Figure 13:**
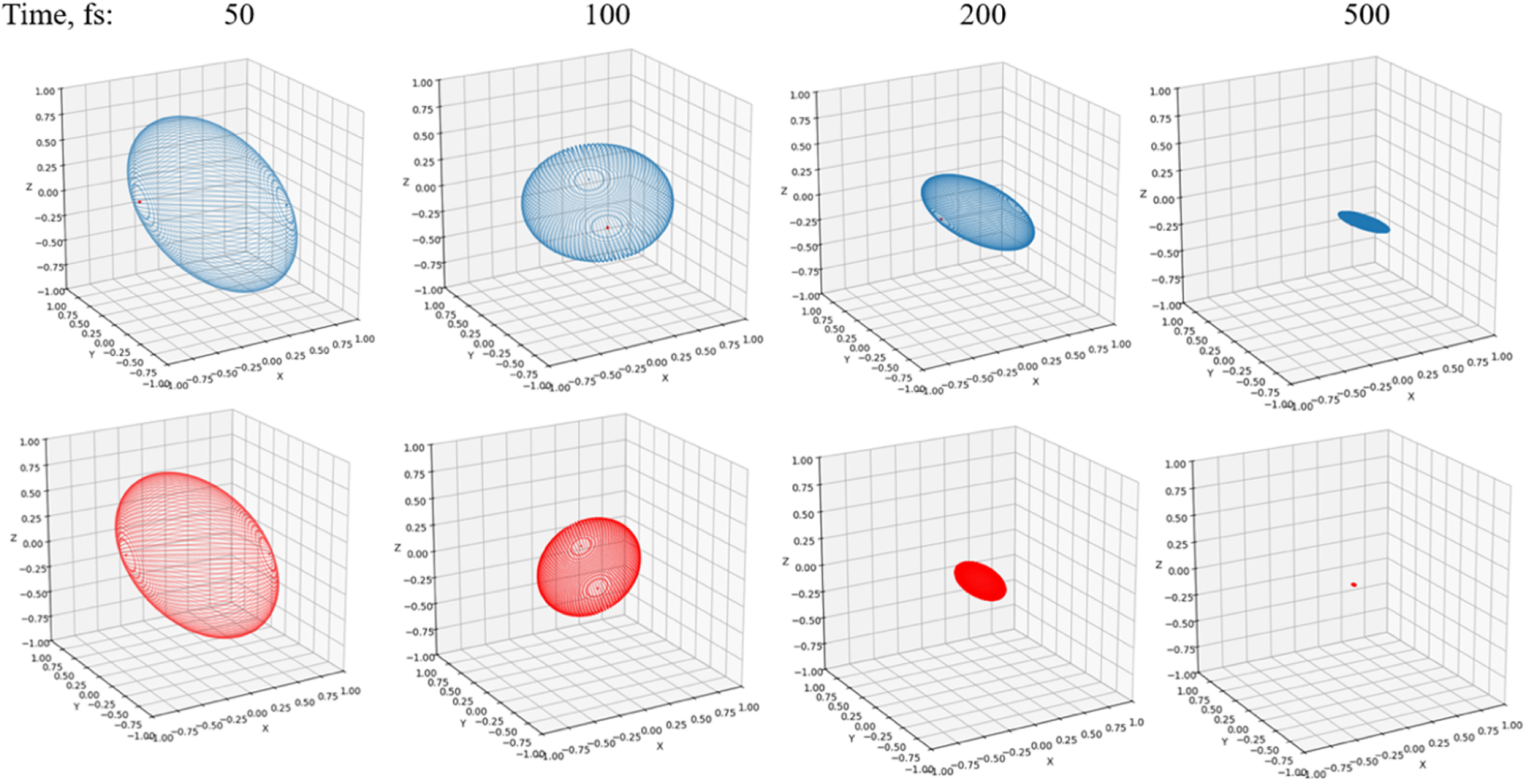
Bloch sphere under non-Markovian (blue) and Markovian (red) evolution at *t* = 50 fs, *t* = 100 fs, *t* = 200 fs, and *t* = 500 fs. Parameters are ℏΩ = 100 cm^–1^ and ℏ*ε* = 50 cm^–1^ for the system and λ = 20 cm^–1^, γ = 53.08 cm^–1^, and *T* = 300 K for the Drude bath.

## 7 Conclusion

In this work, we presented a systematic approach to convert the exact non-Markovian dynamical map to the Lindblad form. The immediate structure of the Lindblad dissipator allows us to extract the decay rates and quantify non-Markovianity. The separation of the negative decay rates enables investigation of the effect of the non-Markovian bath on the properties and the dynamics of the system. We have shown that the non-Markovian bath can better protect coherence and entanglement, change the energy gap, and shift the equilibrium. It is not hard to infer that other properties such as quantum transport and entropy production can be examined in the same way. We have also provided a pictorial representation of the qubit dynamics by the Bloch sphere time evolution and the associated affine transformation. This visualization gives a more direct impression of the dynamic process influenced by the non-Markovian bath. As the extent of non-Markovianity present in the dynamics correlates with the coherence lifetime and state distribution, it is possible to tune the amount of non-Markovianity through spectral density engineering to optimize a particular molecular task. Specifically, with real molecular systems, we can identify the vibrational modes that are important for inducing non-Markovianity and modify the corresponding molecular moiety. We anticipate that the approach we provided will be particularly insightful for investigating multistate quantum dynamics, such as exciton transport in molecular aggregates and proton-coupled electron transfer dynamics, to address the question of how non-Markovianity shapes the reaction pathway and transfer rate. Such an investigation is currently underway in our group.
